# Correction: Abazari, A.M., *et al*. Modelling the Size Effects on the Mechanical Properties of Micro/Nano Structures. *Sensors* 2015, *15*, 28543–28562

**DOI:** 10.3390/s16060781

**Published:** 2016-05-27

**Authors:** Amir Musa Abazari, Seyed Mohsen Safavi, Ghader Rezazadeh, Luis Guillermo Villanueva

**Affiliations:** 1 Department of Mechanical Engineering, Isfahan University of Technology, Isfahan 84156-83111, Iran; mosafavi@cc.iut.ac.ir; 2 Advanced NEMS Group, École Polytechnique Fédérale de Lausanne (EPFL), Lausanne CH-1015, Switzerland; 3 Department of Mechanical Engineering, Urmia University, Urmia 57561-51818, Iran; g.rezazadeh@urmia.ac.ir

The authors wish to make the following correction to this paper [1]: The article type should be changed from “*Review*” into “*Article*”. The authors would like to apologize for any inconvenience caused to the readers by this change.
